# Amygdala Regulation Following fMRI-Neurofeedback without Instructed Strategies

**DOI:** 10.3389/fnhum.2016.00183

**Published:** 2016-04-26

**Authors:** Michael Marxen, Mark J. Jacob, Dirk K. Müller, Stefan Posse, Elena Ackley, Lydia Hellrung, Philipp Riedel, Stephan Bender, Robert Epple, Michael N. Smolka

**Affiliations:** ^1^Department of Psychiatry and Neuroimaging Center, Technische Universität DresdenDresden, Germany; ^2^Department of Neurology, School of Medicine, University of New MexicoAlbuquerque, NM, USA; ^3^Medical Faculty, Department of Child and Adolescent Psychiatry, Psychosomatics and Psychotherapy, University of CologneCologne, Germany

**Keywords:** neurofeedback, amygdala, regulation, real-time fMRI, emotions

## Abstract

Within the field of functional magnetic resonance imaging (fMRI) neurofeedback, most studies provide subjects with instructions or suggest strategies to regulate a particular brain area, while other neuro-/biofeedback approaches often do not. This study is the first to investigate the hypothesis that subjects are able to utilize fMRI neurofeedback to learn to differentially modulate the fMRI signal from the bilateral amygdala congruent with the prescribed regulation direction without an instructed or suggested strategy and apply what they learned even when feedback is no longer available. Thirty-two subjects were included in the analysis. Data were collected at 3 Tesla using blood oxygenation level dependent (BOLD)-sensitivity optimized multi-echo EPI. Based on the mean contrast between up- and down-regulation in the amygdala in a post-training scan without feedback following three neurofeedback sessions, subjects were able to regulate their amygdala congruent with the prescribed directions with a moderate effect size of Cohen’s *d* = 0.43 (95% conf. int. 0.23–0.64). This effect size would be reduced, however, through stricter exclusion criteria for subjects that show alterations in respiration. Regulation capacity was positively correlated with subjective arousal ratings and negatively correlated with agreeableness and susceptibility to anger. A learning effect over the training sessions was only observed with end-of-block feedback (EoBF) but not with continuous feedback (trend). The results confirm the above hypothesis. Further studies are needed to compare effect sizes of regulation capacity for approaches with and without instructed strategies.

## Introduction

If humans could more easily learn to improve self-regulation of particular brain functions or states such as emotions or motor tasks through some kind of training with lasting effects, this could have a large impact on areas such as mental and neurological health care and education. Neurofeedback, providing the subject with information about their own brain activity, has been a prime candidate to achieve this. While neurofeedback based on electroencephalographic (EEG) recordings has been investigated for a number of decades (Budzynski et al., 2009), another type of neurofeedback based on functional magnetic resonance imaging (fMRI) is a much newer technique that has experienced a surge of interest within the neuroimaging community within recent years. Among these studies, target sites involved in emotional reactivity and regulation, processes that our group is particularly interested in, are receiving a lot of attention: amygdala (Posse et al., 2003a; Johnston et al., 2010, 2011; Zotev et al., 2011, 2013, 2014; Paret et al., 2014, 2016; Young et al., 2014; Yuan et al., 2014), insula (Caria et al., 2007, 2010; Eippert et al., 2007; Lee et al., 2011; Veit et al., 2012), and anterior cingulate cortex (ACC; Weiskopf et al., 2003; deCharms et al., 2005; Hamilton et al., 2011).

The vast majority of these studies used instructed or suggested regulation strategies during the training, further on referred to as the “explicit strategy” approach. The most common approach to up-regulate the amygdala uses, for example, happy imagery with an effect size of Cohen’s d ≊ 1 for the amygdala signal change in a transfer run without feedback following three feedback training runs (Zotev et al., 2011). In some cases, the strategy may be regarded as central to the scientific objective of the study, e.g., when happy imagery is considered in the context of treating patients with depression. In other cases, however, it is more motivated by a desire to reduce the chances that subjects may not find any suitable way to regulate. In this case, the experimenters may instruct the subject that they only provide “suggested” strategies but that other strategies are also permitted, see e.g., (Caria et al., 2010; Johnston et al., 2010; Paret et al., 2014). It needs to be considered in this context that most published studies have used only a single scanning session with just a few neurofeedback training runs, which provide little time to explore alternative strategies.

In EEG-based neurofeedback, on the other hand, 10–20 training sessions are common and often no strategies are proposed. Such an approach poses no limitations on regulation ability through prescription of a particular strategy. Participants are free to optimize a personal strategy and may even utilize processes that they cannot describe well in words and that may be executed subconsciously without explicit cognitive effort. Even instructions that only suggest strategies and emphasize the freedom to deviate from this suggestion are likely to influence the space of possible strategies that the subject will consider or, at least, will influence the search behavior within this space. On the other hand, even when concrete strategies are being prescribed, it is possible that subconscious processes such as operant conditioning are key with respect to the learned regulation ability. Leaving subjects room to explore alternative strategies may favor the utilization of subconscious conditioning effects. This may be of particular relevance with respect to the issue of “transfer” addressing the question of whether the training has led to lasting regulation skills that can be utilized without neurofeedback. While conscious strategies may still transfer well when simply eliminating the feedback signal from the training task, subconscious processes may be advantageous when considering transfer to everyday life situations. This could be particularly relevant in the context of emotions which we usually regulate subconsciously.

Currently, we are only aware of one study targeting emotional processes that did not use a suggested or instructed strategy (Johnston et al., 2011) to regulate the amygdala in the absence of emotion-inducing stimuli. In that study, target regions were defined individually based on the highest effect size for a contrast positive vs. neutral pictures from a localizer scan and included ventrolateral prefrontal cortex (vlPFC), dorsolateral PFC (dlPFC), insula, and medial temporal lobe (MTL). Subjects were informed of this localization strategy. While the study could demonstrate regulation success in the experimental group (*n* = 17) in comparison to a control group (*n* = 10) that was looking at a static “thermometer”-display, it is difficult to draw conclusions from this study regarding the ability of subjects to regulate any particular anatomical region successfully. Subjects reported using strategies involving positive memories and imagery to up-regulate their target areas, for example visualizing being with friends and family. They also often reported testing different strategies before settling for one. For the visual system, Shibata et al. (2011) have shown, using a classifier-based approach, that subjects could find ways to enhance sensitivity for particular visual stimuli providing additional evidence that a “no instructed strategy” approach may be effective.

**Figure 1:**
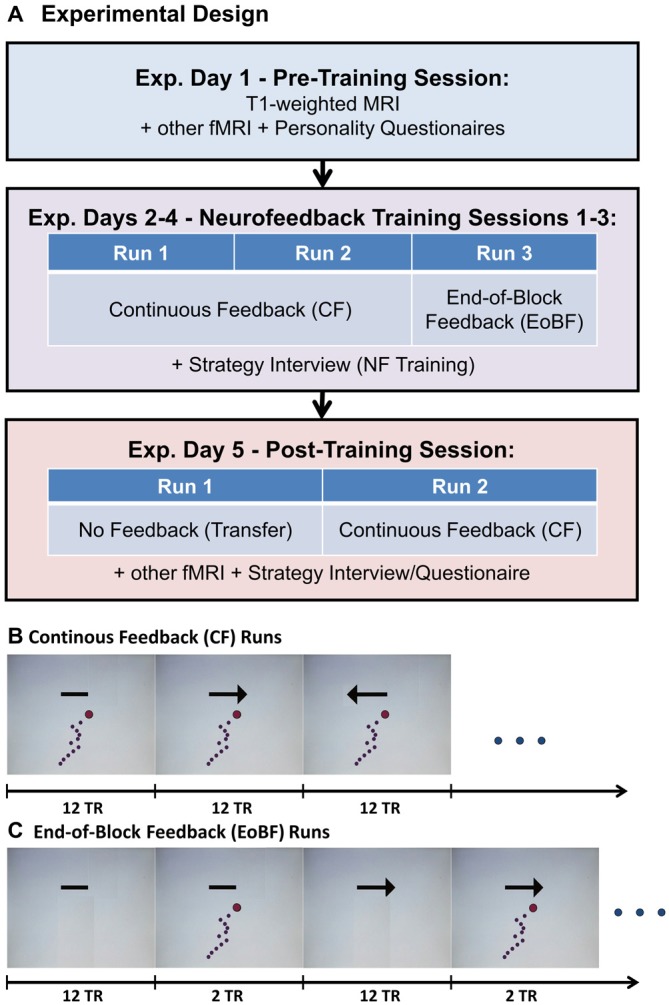
**(A)** Experimental design. Each of the three feedback training sessions consisted of two runs with continuous feedback **(B)** followed by one run with end-of-block feedback (EoBF; **C**). The feedback display, shown in **(B)** and **(C)**, is updated every TR. The arrow indicates the regulation direction, which is linked to “UP”- or “DOWN”-regulation of the amygdala in a counter-balanced fashion. A simple bar without arrow head indicates a rest period. Regulation or rest periods were 12 *TR* ≊ 30 s in length (*TR* = 2.54 s). All runs began with a rest block followed by 8 blocks each of left regulation, right regulation, or rest. The dots mark the amygdala signal from the last 12 fMRI image acquisitions. The red dot represents the most recently acquired data, while the purple dots represent progressively older data.

The goal of the present study was to investigate thoroughly whether participants are able utilize fMRI neurofeedback to learn to regulate the amygdala in congruence with the prescribed regulation direction without any suggested strategies and whether they could employ what they learned after training under transfer conditions without receiving neurofeedback. The driving motivation related to this initial goal was to subsequently study the influence of self-regulated amygdala activity on emotional reactivity. For this purpose, neurofeedback was combined with emotional reactivity tasks in the post-training session. We will report on these experiments elsewhere. In comparison to most published fMRI neurofeedback studies, we are presenting data from a large group of subjects (*N* = 32) using an intensive training period with three training sessions conducted on separate days. To promote transfer of acquired skills to a no-feedback setting, we introduced a training run at the end of a training session with end-of-block feedback (EoBF; see Figure 1). This last run allowed the subjects, once they had found a promising strategy during the initial continuous feedback runs, to focus on practicing this strategy without distraction by the feedback. There is some evidence that EoBF improves regulation ability for instructed strategies (Johnson et al., 2012; Dietrich et al., 2014)^1^. Lastly, as a transfer run directly following the training may not represent lasting regulation abilities in the absence of feedback, we conducted a post-training session on a different day to investigate transfer.

Our primary outcome measure in this study was the difference in the fMRI model regression coefficients for the “UP” and “DOWN” regulation conditions in a no-feedback (transfer) run conducted at the beginning of the post-training session. In our eyes, it is important that this measure is acquired under transfer conditions because otherwise a utility of the training for everyday life, when feedback is not available, could not be demonstrated. For clarity, we will be using the term “regulation capacity” consistently throughout the article to describe this regulation ability under transfer conditions while the term “regulation ability” will be used for all other runs. We also investigated learning curves as a secondary measure of training success. However, we avoid such curves as the primary outcome measure not only because they would ignore transferability in our case but because learning cannot be expected to occur in a linear or even monotonic fashion when subjects are asked to experiment with different strategies. This is an important difference to investigations that focus on improving an instructed regulation strategy.

## Materials and Methods

### Participants

After an initial piloting phase, 35 healthy, right-handed (Edinburgh Handedness Inventory (EHI) score > = 50) adults between the ages of 18–40 years participated in this study. Of these, three were excluded, one because of technical problems, one dropped out for personal reasons, and one was excluded because of obvious disregard of the instructions not to use movement to regulate, resulting in *N* = 32 (15 females, mean age 24.7 years) usable data sets. Exclusion criteria at recruitment included a history of mental disorder, physical conditions that prevent lying comfortably inside an MRI scanner, a body mass larger than 120 kg, vision impairments outside of −5 to +3 diopters, insufficient knowledge of the German language, potential pregnancy, and contra-indications for MR-scanning. The study was approved by the Ethics Review Board of the Technische Universität Dresden. All subjects signed informed consent forms after receiving a detailed description of the experiment. Subjects received approximately €90.00 compensation for participating in this study on the last experimental day.

### Experimental Design

The experimental design is illustrated in Figure 1. The prescreening included the EHI and questions on body mass and size, MR-compatibility, medication, physical and mental disorders, pregnancy, drug consumption, and smoking history. Subjects that had met all inclusion criteria were invited to a pre-training MRI session (Experimental (Exp.) Day 1). During this session, a T1-weighted MRI was acquired, which was used to automatically define a subject-specific, bilateral amygdala target region-of-interest (ROI). Additionally, a resting state fMRI and two fMRI-tasks were acquired, which we are not reporting on here. The first task was designed to measure attentional capture effects on reaction times by emotional picture stimuli, which were temporally flanking a simple reaction time task. The second task was a fear conditioning task designed to measure the effect of fear on reaction time. The subjects also filled out a number of personality questionnaires (see “Questionnaires and Interviews” Section) and were familiarized with the feedback display and the regulation instructions (see “Feedback Display and Regulation Instructions” Section and “Supplementary Material S1”). Subjects were also given the opportunity to experience the hemodynamic delay present in fMRI as they manipulated the feedback display via button presses that only affected the display with a delay of 6 s. Subsequently, participants were scheduled for four scanning sessions including three identical training sessions and a post-training session, which included a transfer test. These sessions were scheduled ideally with 1–3 days in between but some flexibility was required to adapt to scanner availability, technical difficulties, and no-shows.

A training session consisted of three training runs in total: 2 runs of 12:42 min. each with continuously (every TR) updated feedback (CF) and a third run of 14:49 min. with EoBF only (see Figures 1B,C). Each run began with a rest block followed by eight blocks of left regulation, right regulation, or rest. In approximately half of the subjects, “left” corresponded to an up-regulation of the amygdala, in the other half to a down-regulation (see “Target ROI Definition and Real-Time fMRI Data Analysis” Section). A single block was 12 TR = 30.48 s long. Block order was pseudo-randomized such that conditions always change and each of the six possible block transition types (e.g., left-regulation followed by rest) occurs four times. The post-training session (Exp. day 5) began with a transfer run without feedback (same display as in Figure 1B but without the dots) followed by a conventional regulation run with feedback. Following this, two fMRI tasks similar to the tasks from day 1, but this time combined with neurofeedback, were conducted to test the effect of amygdala regulation on these tasks. Results of these experiments will be reported elsewhere.

### Feedback Display and Regulation Instructions

We decided to use a feedback display (see Figure 1) that shows the fMRI signal from the previous 30 s in form of horizontally moving dots because, in this way, the display is not as susceptible to noisy, single measurements and subjects will easily notice whether they regulate successfully through comparison with the signal history. This approach is similar to the scrolling graph of MRI data, which was employed by Hamilton et al. (2011). The regulation instructions are presented to the subject in writing together with a picture of the feedback screen (see Figures 1B,C). The text of the instructions with an English translation is available in “Supplementary Material S1”. Briefly, the instruction was to move the signal (the dots) in the direction indicated through a purely mental strategy. Subjects were asked not to move, to breathe normally, and to keep the eyes open. They were also informed that their goal should be to develop, after some try outs, a single favorite strategy for each regulation direction that they would be asked to employ in an additional session without feedback (the transfer run in the post-training session).

### Questionnaires and Interviews

On day 1, subjects completed German versions of the following personality questionnaires: NEO-Five Factor Inventory (FFI; Costa and Mccrae, 1992), State-Trait-Anxiety Inventory (STAI; Kendall et al., 1976), Emotional Regulation Questionnaire (Gross and John, 2003), Emotional Contagion Scale (ECS; Doherty, 1997), Toronto Alexithymia Scale (TAS-20; Bagby et al., 1994), Perceived Stress Scale (PSS; Cohen et al., 1983), Beck Depression Inventory (BDI II; Beck et al., 1996).

After each training session, a standardized interview was conducted primarily with respect to employed strategies. The interviewer asked what strategies the subject had tried during this session, how successful they judged these strategies on a 5-point scale, and whether they would prefer continuous or EoBF in another training session (part A). The last question (preference-for-feedback type) was of no consequence for the actual feedback type employed in the following session. The subjects were also asked (part B) to rate on 5-point scales their performance at the end of the session, their belief whether they would be able to improve, and questions related to their well-being during the session. No hints on possible strategies were given. Following the last session (5), an interview was conducted similar to part B above but requesting the information specifically for each experimental run of that day. Additionally, the subjects were asked to fill out a questionnaire on their best strategies for left- and right-regulation (see “Supplementary Material S3”). For presentation purposes, the data was reassigned to the conditions of interest: “UP” or “DOWN”. The questionnaire attempts to assign 1 of 4 categories to each strategy: (1) Focusing on something in the present moment (e.g., breathing); (2) Imagination (e.g., happy memories) – For this category, subjects were also asked to specify whether such imaginations involved: (a) visual scenes; (b) sounds/music/speech; (c) smell; (d) movements; (e) taste; or (f) touch; (3) Abstract thoughts (e.g., mental arithmetic); (4) Bodily strategies (e.g., breath manipulation). Category four was a check as to whether subjects followed the regulation instructions. They were also asked to rate their arousal and valence during the regulation conditions on a 5-point scale using Self-Assessment-Manikin (SAM) images (Lang, 1980).

### fMRI Data Acquisition

Images were acquired on a 3-Tesla Siemens Tim Trio scanner using the Siemens 32-channel head coil (Siemens, Erlangen, Germany). T1-weighted images were acquired with a 3D magnetization-prepared rapid gradient echo (MP-RAGE) sequence (repetition time (TR) = 1.9 s, echo time (TE) = 2.26 s, field of view (FOV) = 256 × 224 × 176 mm^3^, voxel size = 1 × 1 × 1 mm^3^, inversion time = 0.9 s, flip angle (FA) = 9°, phase partial Fourier 7/8, bandwidth (BW) = 200 Hz/Px). To maximize blood oxygenation level dependent (BOLD) sensitivity in the amygdala, which suffers considerable susceptibility-related signal losses, we employed a multi-echo EPI sequence and online ${\text{T}}_{2}^{*}$-weighted echo averaging optimized for the amygdala as described in our previous studies (Posse et al., 1999, 2003a). Functional data were acquired with six echoes (TR = 2.54 s, TE = 8.6, 18.3, 28, 38, 48, 57 ms, FOV = 192 × 192 × 132 mm^3^, voxel size = 4 × 4 × 3.2 mm^3^ with a slice gap of 25%, GRAPPA with ipat factor three and 42 reference lines, FA = 82°, BW = 2084 Hz/Px, slice orientation A > C, slice order: descending). The parallel imaging acceleration was chosen to minimize geometrical distortion in the amygdala due to susceptibility inhomogeneity without incurring significant increase in parallel imaging related noise enhancement. EPI images were distortion-corrected in real-time based on point spread function mapping using a phase-encoded prescan with a single TE = 8.7 ms (Zaitsev et al., 2004). The physiological parameters heartbeat and respiration were recorded using the Siemens pulse plethysmograph and respiratory belt.

### Target ROI Definition and Real-Time fMRI Data Analysis

The bilateral amygdala was selected as the target region for this study because both sides of the amygdala are usually highly correlated during emotional reactivity tasks (Mitchell et al., 2008) and because we observed in pilot studies a bilateral activation of the amygdala for the emotional reactivity task that we integrated in this study. To extract individual target regions, the T1-weighted image from session 1 was co-registered to a weighted EPI-image from the same session (MoCo-target, see below) using SPM8^2^. Subsequently, the T1-image was parcellated into anatomical regions using Freesurfer’s (Dale et al., 1999; Fischl et al., 2002, 2004) recon-all version 1.379.2.73. A mask image of the bilateral amygdala was generated in the space of the MoCo-target to define the neurofeedback target region.

To generate the neurofeedback signal, reconstructed and distortion-corrected images were exported directly from the Siemens reconstruction computer via a custom designed TCP/IP-based pipeline to the scanner console and forwarded to an external Linux workstation (Intel(R) Core(TM) i7–3770K CPU @ 3.50 GHz, 16 GB RAM, 1 TB SSD) running TurboFire Version 5.14.4.0 (NeurInsight LLC, Albuquerque, NM, USA; Gembris et al., 2000) inside a virtual Linux machine (1 CPU, 10 GB RAM). TurboFire performed motion correction of the weighted images to the MoCo-target generated in the pre-training session. Multi-echo images were combined using fixed TE-dependent weights 0.59, 0.90, 1, 0.97, 0.88, 0.77, which were selected for an average ${\text{T}}_{2}^{*}$-value of 30 ms in the amygdala (Posse et al., 1999, 2003b). The fixed weights in this analysis minimized possible fluctuations due to instability in ${\text{T}}_{2}^{*}$ fitting during the real-time scan, while only slightly reducing the maximum possible BOLD sensitivity in the rest of the brain. The mean signal in the target ROI was written to a text file, which was read by a Python script, running on the host of the virtual machine, that scaled the signal, and automatically counterbalanced whether regulation to the right on the screen corresponds to up or down regulation of the amygdala. This information was also logged in a text file. Image volumes 6–15 from each run, always acquired during the “REST” condition were used to calculate the mean and standard deviation of the signal. The mean was used as a baseline and subtracted from all following signal values. A signal equal to the mean placed the corresponding dot at the center of the display, while an offset of 4 standard deviations corresponds to the edge of the display. Prior to each run the scaling was automatically reset to account for scanner drift. During a single run, no drift correction was necessary because the dots would stay within the screen. Feedback dot positions were written into a text file on a drive that was shared with another computer running Presentation 16.3. (Neurobehavioral Systems Inc., Berkeley, CA, USA), which generated the feedback screen (see Figures 1B,C).

### Offline Data Analysis

The fMRI data were preprocessed using a random effects model and SPM8 (Wellcome Department of Cognitive Neurology, London, UK) including slice-time correction, realignment (motion correction), T1-based normalization, and smoothing with an 8 mm FWHM kernel. For a whole brain, subject-level analysis, a general linear model (GLM) with two regressors for “UP”- and “DOWN”-regulation convolved with SPMs canonical hemodynamic response function (HRF) and six motion regressors was estimated for the continuous feedback runs and the no-feedback transfer run. For the EoBF runs, a third regressor for the feedback display was included. The data was high-pass filtered at a frequency of 1/128 Hz. The “REST” condition served as the implicit baseline. The regression coefficients of interest (beta_oi) were transformed to percent signal change values (PSC) using the equation PSC = (beta_oi*max(Single Event convolved with HRF) * 100)/beta_constant, where beta_constant is the coefficient of the constant (=1) GLM term. On the group level, a full-factorial model with one factor and two levels (“UP” and “DOWN”) was computed.

A whole-brain analysis with this model was only conducted to illustrate the distribution of *t*-values for the “UP-DOWN” contrast throughout the brain, which should show clusters of higher values in the amygdala region with respect to the rest of the brain. Clusters throughout the brain or extending over large areas of the brain would indicate that the employed regulation mechanism was not region-specific. To illustrate the cluster distribution, a *p*-value threshold of 0.05 uncorrected for multiple comparisons was used.

To evaluate regulation success, each subject’s amygdala mask was co-registered to the subject’s slicetime- and motion-corrected EPI data using SPM8, and the mean regression coefficients (PSC) from the bilateral amygdala were extracted. 1-tailed *t*-tests for each contrast (“UP-DOWN”, “UP-REST”, and “REST-DOWN”) > 0 were computed on the group level using Matlab (The Mathworks, Inc., Natick, MA, USA). *P*-values uncorrected for multiple comparisons are quoted for all runs. We will conduct specific hypothesis tests (see “Introduction” Section) focusing on the transfer run and learning rates. Corrections for multiple comparisons, which would be required for an exploratory analysis, are not employed. A significant (*α* = 0.05) contrast “UP-DOWN” > 0 for the transfer run without feedback was taken as evidence that subjects could regulate successfully after the training without feedback. The effect size was computed as Cohen’s d = (group mean)/(group standard deviation). The 95% confidence interval was estimated using bootstrapping with 1000 draws. Data is presented for *N* = 32 subjects with the qualification that four runs were not analyzed due to technical difficulties (2 EoBFs of training day (TD) 1, 1 EoBF of TD 3, and the 2nd CF of TD 1). The reduced degrees of freedom were accounted for in the statistics.

To investigate possible differences between conditions in heart rate (HR) or breathing patterns in the transfer run, we used the PhysIO toolbox^3^ (Kasper et al., 2009) to extract timecourses of HR and respiration volume per time (RVT; Birn et al., 2008) from the recorded physiological waveforms. RVT can be regarded as a measure of how much air is passing through the lungs per time. Means of these parameters were computed for the “UP”, “DOWN”, and “REST” conditions and analyzed using a 3-level repeated-measures analysis of variance (ANOVA). To compute Pearson correlations with regulation capacity, relative “UP”-“DOWN” difference with respect to the “REST” conditions in these parameters were computed.

To investigate learning rates and their interactions with feedback type and regulation direction, we computed a 3 × 2 × 2 repeated-measures ANOVA using SPSS23 (IBM Corp., Armonk, NY, USA) with the factors of “TD” (1–3), “Feedback Type” (“CF” vs. “EoBF”), and “Regulation Direction” (“UP” vs. “DOWN”). For this analysis the two continuous runs were averaged. A possible predictive correlation between regulation capacity (“UP-DOWN” contrast in the transfer run) and the last training run with EoBF was tested using Pearson correlation. The same approach was taken to test a possible correlation between regulation capacity and regulation ability in the post-training continuous feedback run. To validate our assumption that the association of increasing BOLD signals with left or right moving dots would have no effect on regulation capacity, we conducted a single factor ANOVA.

Personality questionnaires were mostly used in an exploratory fashion using a Bonferroni correction for significance. Pearson correlations of questionnaire scores with regulation capacity were computed. Two specific hypotheses based on findings by Zotev et al. (2011) were tested: that (a) regulation capacity is negatively correlated with the Difficulty Identifying Feelings sub-score of the TAS-20; and (b) negatively correlated with the Susceptibility to Anger sub-score of the ECS. A hypothesized positive correlation of regulation capacity with self-evaluation scores for the post-training runs was tested using Pearson correlation. Similarly, we tested whether the arousal rating difference between “UP” and “DOWN” regulation conditions was correlated with regulation capacity. The preference-for-feedback type question was analyzed using repeated-measures ANOVA with the factor training session and a subsequent one-sample *t*-test against no preference (0 – preference for CF, 1 – for EoBF, 0.5 – no preference).

A list of reported strategies judged as most successful is given for the eight best (25%) regulators based on their subject-level amygdala ROI median *p*-value of the up-down contrast. We will also provide a summary table of the subjective strategy categorization for all subjects in a supplement. We have condensed this information here somewhat because it was not a primary objective of this study to identify working strategies (see “Discussion” Section).

## Results

### Regulation Effects in the Amygdala

**Figure 2:**
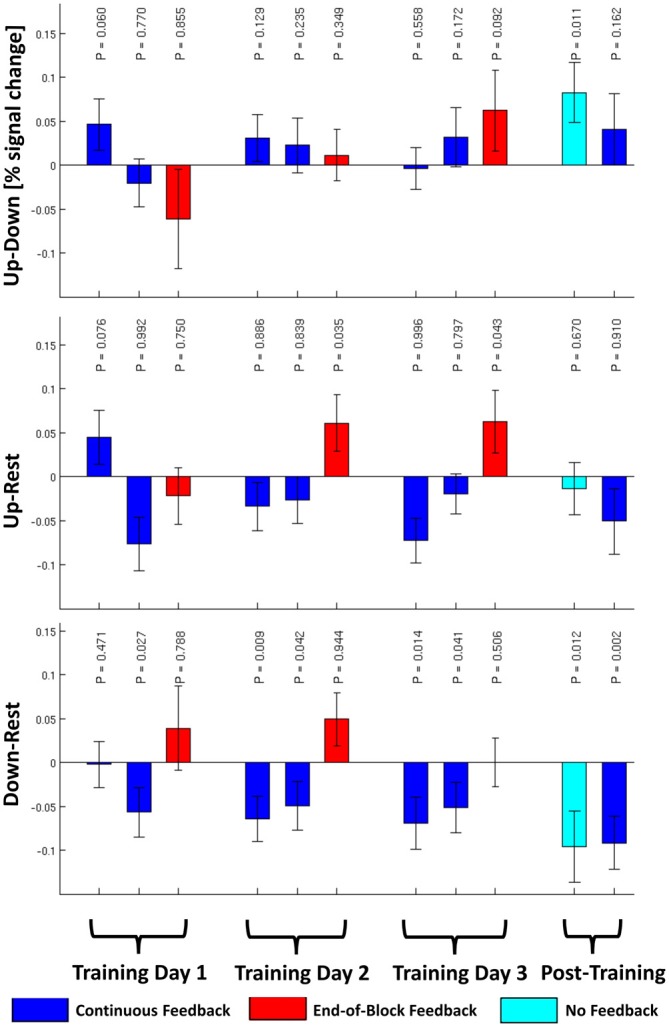
**The group averaged contrasts for “UP” against “DOWN”, “UP” against “REST”, and “DOWN” against “REST” conditions are plotted for each of the 11 runs (±1 SEM) for *N* = 32 subjects.** *P*-values uncorrected for multiple comparisons for the 1-tailed, one-sample *t*-test are given using the alternative hypotheses that “UP” > “DOWN”, “UP” > “REST”, and “DOWN” < “REST”.

The primary hypothesis of this article that subjects would be able to regulate their amygdala volitionally in the transfer run without receiving strategy instructions in the training was confirmed. The estimated BOLD signal in the “UP” regulation condition was significantly higher than in the “DOWN” regulation condition in the no-feedback transfer run (*p* = 0.011, see Figure 2). The effect size in the transfer run was Cohen’s d = 0.43 (95% conf. int. 0.23 − 0.64). None of the other runs in Figure 2 show a significant effect for the “UP-DOWN”-contrasts. The estimated signal changes for the “UP” and “DOWN” regulation conditions compared to the “REST” condition are also shown in Figure 2. None of them is significant when correcting for multiple comparisons (Bonferroni Correction for 22 runs α=0.05/22 = 0.002) with the borderline exception of the “DOWN-REST”-contrast in the post training continuous feedback run.

With respect to learning curves, the 3 × 2 × 2 ANOVA of the training runs with the within-subject factors “TD” (1–3), “Feedback Type” (“CF” or “EoBF”), and “Regulation Condition” (“UP” or “DOWN”) resulted in a main effect of the factor “Feedback Type” (Tests of intra-subject contrasts: *F*_(1,27)_ = 7.7, *P* = 0.010, *N* = 28, 4 of 32 subjects were not included in this analysis because of incomplete data). This can be explained by a generally higher activation during regulation (“UP” or “DOWN”) in the EoBF condition with respect to “REST” than for the CF condition (see Figure 2). Beyond this, only the 3-way interaction of the linear effects of all factors showed a trend (Tests of intra-subject contrasts: *F*_(1,27)_ = 3.3, *P* = 0.081). This can be interpreted as a result of the slope of the training-day effect being higher for “UP” regulation than for “DOWN” regulation but only in the EoBF condition. In other words, the subjects” ability to regulate differentially during the “UP”- and “DOWN”-conditions improves over sessions but only when EoBF is presented, not with continuous feedback, consistent with the assumption that subjects are trying out different strategies with variable success during the CF runs.

**Figure 3:**
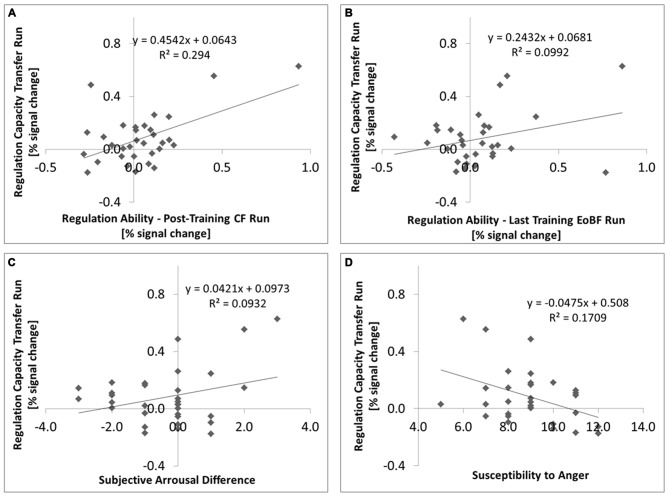
**Individual regulation capacity, which refers throughout the manuscript to the BOLD model contrast “UP” against “DOWN” in the post-training transfer run, is plotted as a function of various parameters: (A) vs. regulation ability in the post-training continuous feedback run (*N* = 32, *P* = 0.001); (B) vs. regulation ability in the EoBF run of the last (3rd) training session (*N* = 31, *P* = 0.042); (C) vs. the difference in subjective arousal ratings between the up-regulation and down-regulation conditions for the post-training session (*N* = 32, *P* = 0.045); **(D)** vs. the Emotional Contagion “Susceptibility to Anger” subscale (*N* = 31, *P* = 0.019).** Fitting coefficients and the coefficients of determination are given.

Regulation capacity (transfer run) was positively correlated with regulation ability in the following continuous feedback run (1-tailed Pearson *R* = 0.542; *P* = 0.001, *N* = 32, Figure 3A) despite the fact that the group effect of regulation ability in the post-training CR run was not significant. Regulation ability in the last EoBF run of TD 3 was predictive of regulation capacity (1-tailed Pearson *R* = 0.315; *P* = 0.042, *N* = 31, Figure 3B) as was, at a trend level, the slope of regulation abilities in the EoBF runs over all three TDs (1-tailed Pearson *R* = 0.303; *P* = 0.055, *N* = 29). We confirmed that there was no effect of the regulation direction assignment (e.g., “UP” on “LEFT”) on regulation capacity (*F*_(1,30)_ = 0.904; *P* = 0.349).

### Whole Brain Analysis

**Figure 4:**
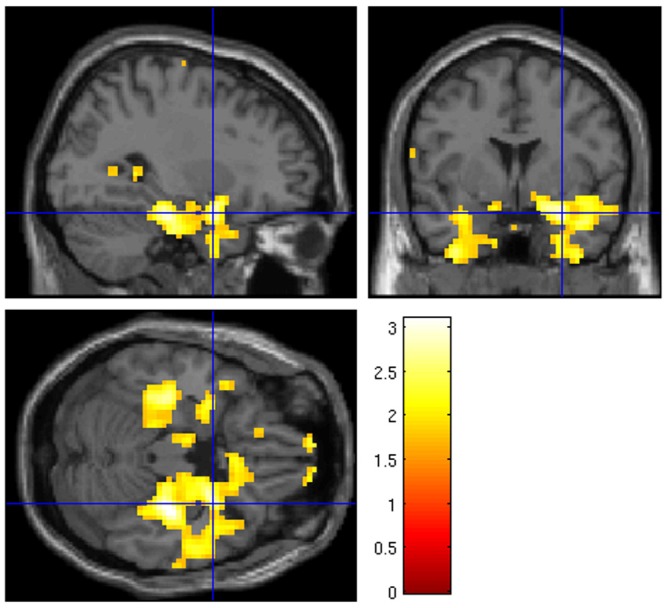
**Whole brain group analysis of the contrast “UP” against “DOWN” regulation at *p* < 0.05 uncor.** The blue crosshairs mark the right amygdala MNI coordinate [30, 2, −23]. The scale represents *t*-values (*df* = 31).

**Figure 5:**
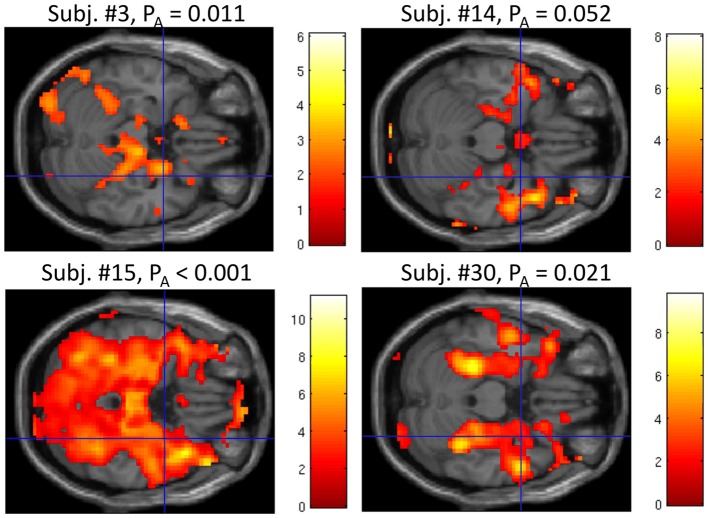
**Whole brain analysis of the contrast “UP” vs. “DOWN” regulation at *P* < 0.05 uncor. for the four “best” regulators based on the median *P*-values within the amygdala P_A_.** The blue crosshairs mark the same position as in Figure 4 in the right amygdala (MNI coordinate [30, 2, −23]). The scale represents *t*-values (*df* = 280). While the activation in subjects 3, 14, and 30 does not appear to be driven by motion or physiology, in subject 15, it may be related to alterations of breathing, which could have induced a widespread increase of the BOLD signal.

At liberal thresholds (*P* < 0.05 uncor.), local maxima in the group *t*-map for the “UP-DOWN” contrast can be identified in the right amygdala (MNS coordinate [30, 2, −23], *t* = 3.03) and very near the left amygdala ([−33, −1, −23], *t* = 2.49; see Figure 4). At a more conservative threshold of *p* < 0.001 (uncorr.), no activation was found. These activations do not survive corrections for multiple comparisons, which is not surprising given the effect size reported above. Applying a small volume correction for the bilateral amygdala, the FWE-corrected peak *p*-value reaches 0.054 and is located in the right amygdala. However, we are showing these images here only to illustrate the pattern of activation, which indicates that the activation is regionally specific and not a whole brain effect. Whether regulation was driven by physiological changes, however, is better judged on the subject-level. For this purpose, we are presenting subject-level activation maps for the four best regulators in Figure 5. While the activation maps for subjects 3, 14, and 30 do not appear to be a result of motion or physiological artifacts, the map for subject 15 shows widespread BOLD activation, which may be a result of a change in respiration.

### Physiological Correlates

**Figure 6:**
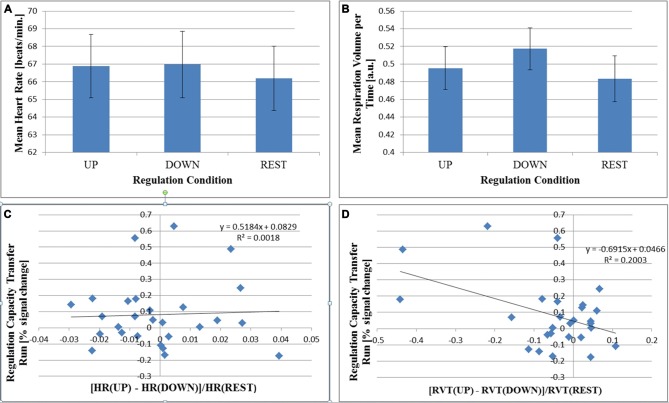
**Group mean values for heart rate (HR; A) and respiration volume per time (RVT; B) are shown for each regulation condition during the no-feedback transfer run (*N* = 27; ±EOM).** There is a significant within-subject effect of regulation condition for HR (*P* = 0.004) driven by the difference between both “UP” and “DOWN” conditions with respect to “REST”. There is no significant difference between “UP” and “DOWN” conditions. For RVT, there is only a trend for a difference between conditions (*P* = 0.051). In **(C,D)**, regulation capacity in the no-feedback transfer run is plotted as a function of the relative difference between “Up” and “DOWN” conditions in HR **(C)** and in RVT **(D)**. The Pearson correlation is significant for RVT (*P* = 0.018, **D**) but not for HR (*P* = 0.853, **C**).

Effects of regulation condition on HR and RVT for the transfer run are shown in Figure 6. Acceptable physiological recordings were available in 27 subjects (no loss of signal or excessive signal plateaus). We observed an intra-subject effect of regulation condition on HR (*F*_(2,52)_ = 6.092; *p* = 0.004), which was driven by an increased HR during the active (“UP” and “DOWN”) regulation conditions (Figure 6A). The relative difference between “UP” and “DOWN” conditions was not correlated with regulation capacity (2-tailed Pearson *R* = 0.038, *p* = 0.853, Figure 6C). For RVT, there was a trend for an intra-subject effect of regulation condition (*F*_(2,52)_ = 3.148; *p* = 0.051, Figure 6B). Additionally, we found a correlation between the relative RVT-difference between the “UP” and “DOWN” conditions and regulation capacity (2-tailed Pearson *R* = −0.450, *p* = 0.018, Figure 6D). A one-sample *t*-test of the relative parameter differences between the “UP” and “DOWN” conditions results in no significant difference from zero for relative HR (*t*_(26)_ = −0.217, *p* = 0.830 uncor., 2-tailed) and a trend for relative RVT (*t*_(26)_ = −236, *p* = 0.052 (uncor., 2-tailed).

### Questionnaire Results

Because not all subjects completed all questionnaires, we always quote the number of subjects analyzed below. There was a trend for a correlation between the subjects’ perception of their own regulation capacity with the measured effect in the post-training CF run (Pearson *R* = 0.275; 1-tailed *p* = 0.074; *N* = 29) but not for the preceding transfer run (*R* = −0.123; 1-tailed *p* = 0.263; *N* = 29). The difference in subjective arousal ratings between the “UP” and “DOWN” conditions for the subjects best strategies was positively correlated with regulation capacity in the transfer run (Pearson *R* = 0.305; 1-tailed *p* = 0.045, *N* = 32, Figure 3C).

Against our hypothesis, the TAS-20 “Difficulty Identifying Feelings” sub-score did not correlate negatively with regulation capacity. Correlation values were actually positive but not significant: in the no-feedback transfer run: Pearson *R* = 0.144; 1-tailed *p* = 0.219; in the CF post-training run: *R* = 0.191; 1-tailed *p* = 0.151, *N* = 31. The “Susceptibility to Anger” sub-score of the ECS, however, was negatively correlated with regulation capacity in both runs (no feedback: *R* = −0.413; 1-tailed *p* = 0.010; CF: *R* = −0.376; 1-tailed *p* = 0.019, *N* = 31, Figure 3D). An exploratory correlation analysis of regulation capacity with 21 scores from the available personality questionnaires resulted in only one significant finding (Bonferroni Correction α = 0.05/21 = 0.002): The NEO-FFI subscore on “Agreeableness” showed a negative correlation (*R* = −0.539; 2-tailed *p* = 0.002, *N* = 31), meaning that less agreeable/compassionate/understanding people could regulate better.

In the analysis of the preference-for-feedback type question, no effect of session was found and thus the preference scores were averaged over sessions. A 2-tailed, single sample *t*-test against no preference showed a significant preference in our group for CF runs (*t*_(27)_ = −3.146, *p* = 0.004, mean = 0.304, *N* = 28).

When participants were asked at the end of the study whether they had received any other hints with respect to applicable regulation strategies beyond the written instructions, all participants reported they had not. Strategies of the eight most significant regulators are reported in Table 1. Reported strategy categories are provided for all subjects in “Supplementary Table S3”. Strategies across subjects show considerable inter-individual differences. A link between the reported strategies and the regulation direction based on the assumption that the amygdala signal corresponds to arousal is in many cases not obvious.

**Table 1:** **Summary of regulation strategies judged as most successful by the eight best regulators (highest subject-level median amygdala *P*-values for the contrast “UP-DOWN”)**.

Subject #	Regulation capacity [% signal change]/*P*-value	Up-Regulation strategy	Down-Regulation strategy
2	0.245/0.074	I: “kiss, friends, family”; Visual scenes, Body movements; A: “Calculations (no success)”	I: “Death of related people, loss, unpleasant pictures from Session 1”; Visual scenes, Body movements
3	0.555/0.011	CoP: “All happy and sad things that strongly engage me”; I: Visual scenes, Smell; “Relaxing scenes of the past”; B: “not on purpose, but when I had to breathe deeply, movement to the left followed”	A: “difficult calculations”
14	0.179/0.052	CoP: “Breathing”; I:Visual scenes, Sounds/Music/Voices, “moments of happiness, nice memories”; B: “relaxation, breathing”	CoP: “heartbeat”, I: Visual scenes, Sounds/Music/Voices, “sad, stressful moments”; B: “sharp breathing, relaxation”
15	0.628/	#x0003C; 0.001	I:Visual scenes, Sounds/Music/Voices,Body Motions, “situation in which there”s trouble, exaggeratedly imagined”; B: “tension, trouble”	CoP: “relaxation, breathing”; I: Visual scenes, “holidays, relaxation”; B: “relaxation, sometimes lightly holding breath”
16	0.166/0.070	CoP: “solely the dots”; I: Visual scenes, Smell, Touch Sensations, “memories of happy moments, pleasant situations”; A: “counting down, calculating, plan the day”	CoP: “breathing”, I: Visual scenes, Sounds/Music/Voices/, Body Motions, “memories of stressful, sad moments”; A: “form sentences in another language”
24	0.487/0.062	CoP: “counting in French/Italian”; A: “counting upwards, strong concentration”	B: “Neck”
25	0.146/0.087	I: Visual scenes, Body movements, Music, “positive memories (family, friends), specific situations, hiking”	I: Speech “Vocabulary in a foreign language (English und Finnish)”; A: “Math problems, complex multiplications, imagined numbers”
30	0.261/0.021	I: Visual scenes, Sounds/Music/Voices; “walking through familiar buildings, numbers dancing”	O: “multiplication of numbers smaller 100”

*The data is taken from a questionnaire (see “Supplementary Material S2”) that was filled out by the subjects on the last study day after fMRI scanning. (CoP—Concentration on the present, I—Imagination, B—Bodily strategies, A—Abstract thoughts, O—Others)*.

## Discussion

This study confirms that subjects were able to utilize fMRI neurofeedback to learn to regulate the BOLD signal from their bilateral amygdala in prescribed directions without receiving specific regulation instructions or suggested strategies. This conclusion is based on the post-training transfer run without feedback, which showed that amygdala activity was higher in the “UP”-regulation condition than in the “DOWN”-regulation condition. In both active regulation conditions, the amygdala signal was reduced with respect to the baseline condition, which may indicate that regulation effort generally decreases activity in the amygdala. Another factor however, seems to be the nature of the rest condition (see discussion of session effects below).

In the design phase of our study, we carefully considered the need for control conditions or groups to arrive at the above conclusion. Thinking of interventional drug trials, two questions arise: (1) Can we show a difference between the post-intervention outcome measure and the pre-intervention measurement to show that there was an effect of the intervention at all? (2) Can we demonstrate that an observed effect is actually due to the neurofeedback and not due to some unknown learning mechanism that doesn’t require neurofeedback? In the logic of drug trials, question 1 would require a pre-intervention measurement of the outcome variable^4^ and question 2 a placebo control group. However when considering these options, we realized that neurofeedback is fundamentally different from a drug intervention in that it can provide an internal control that eliminates the need for an additional pre-interventional measurement or a control group.

In this regard, the balanced assignment of the “UP” direction to the left- or right-regulation direction is crucial here to control for effects that are unrelated to the feedback. Suppose there were a pre-training or feedback-independent ability of the subjects to bias amygdala activity such that the left-minus-right contrast would be larger than zero, an effect that we cannot exclude. The balanced assignment of left-regulation to the “UP” or “DOWN” regulation conditions would guarantee that the effect on the “UP” minus “DOWN” contrast would be positive in half of the group and negative in the other half. Therefore, an overall group effect can be excluded unless the subjects use the information provided in the neurofeedback signal about what to do in the left-regulation condition vs. the right-regulation condition. It should be noted here that the balanced assignment of “left” onto “UP”/“DOWN” is done by a computer script, which only flips the sign of the signal passed on to the display program, and is unknown to the experimenter. Such a control mechanism is not available in drug trials because it would be equivalent to using an “anti-drug” instead of a placebo that would cause the exact opposite effect on the outcome variable than the “real drug” if any.

It should also be noted that this logic only applies to the “UP” vs. “DOWN” contrast because it is completely symmetric between the two assignment sub-groups but not to the “UP”/ “DOWN” vs. “REST” contrasts. For example, an effect that both active regulation conditions result in a lower amygdala activity as compared to the “REST” condition may be explainable even without neurofeedback. Fortunately, these contrasts individually aren’t good outcome measures for our study in the first place because they may not result in a contrast between the “UP” and “DOWN” conditions. Without such a contrast, directional regulation abilities could not be demonstrated. However, this does not mean that the “REST” conditions is useless for the analysis of the data because it provides important information on the regulation ability with respect to a resting condition once the presence of an “UP” minus “DOWN” effect has been shown.

A study without strategy suggestions is always somewhat vulnerable to the criticism that subjects may have received unintended regulation cues, for example through the nature of the fMRI paradigms in session 1. We consider this unlikely given that none of the subjects made any reference to these tasks when asked at the end of the study whether they had received any information regarding regulation strategies beyond the written instructions. It should be noted, however, following the argument above, that even with a suggested strategy, subjects would have to utilize the neurofeedback information to produce an effect on our primary outcome measure as long as the suggestions are not specific for the “UP” and “DOWN” regulation conditions. Therefore, observing an effect proves that neurofeedback was utilized. The size of the effect, however, may be inflated. To promote unconscious regulation processes, one may consider to avoid the term “mental strategy” completely in favor of an instruction such as “Please, try to move the balls to the right”. However, such an instruction is hard to modify properly for the transfer run. In our case, we want subjects to be able to employ the conscious dimension of their mind as well as the subconscious dimension, which is what differentiates our study from a conventional conditioning experiment.

While individual training runs did not show a significant regulation effect, the success in the transfer run was even observed despite the fact that the transfer test was conducted on a separate day without an opportunity to practice before the test on this day. When clinical applications of neurofeedback are considered, a skill transfer beyond the training period needs to be demonstrated. Thus, our measure of transfer is an advance over previous studies that have conducted amygdala neurofeedback and transfer tests within a single session (Zotev et al., 2011; Paret et al., 2014; Young et al., 2014).

From the analysis of the training sessions and Figure 2, a main effect of feedback type with a generally larger increase of amygdala signals with respect to the resting condition in the EoBF runs is obvious and a higher learning rate in EoBF runs is suggested as compared to CF runs. Additionally, it appears that subjects were only able to up-regulate from baseline in EoBF runs. However, this effect is confounded by the constant order of CF and EoBF runs in each session and also by the difference in the resting baselines between the two feedback types. In the CF runs, subjects were still watching the dots moving on the screen, while no dots were shown in EoBF runs except for 2 TRs at the end of the block. Thus it is possible that amygdala activity may have been higher in the baseline of CF runs compared to EoBF runs, which could have resulted in reduced signals with respect to baseline in the CF runs. A higher learning rate in EoBF runs may be related to subjects trying out different strategies during CF runs at the beginning of each session while focusing more on a particular, promising strategy during the EoBF runs. While our data would be consistent with the claim by others (Johnson et al., 2012; Dietrich et al., 2014) that EoBF can improve learning rates for fMRI neurofeedback when regulation strategies are given, it should not be seen as evidence for such a claim for the above reasons. Our subjects actually preferred the continuous feedback possibly because they had a better sense of how good they were doing in this condition. This would be consistent with the trend correlation between self-evaluated and actual regulation ability in the post-training CF run, which was not seen for the no-feedback transfer run. This remains speculative, however, as there was absolutely no feedback during the transfer run while EoBF runs did supply a feedback. Our decisions with respect to the used type of feedback were governed by assumptions that CFs would benefit the identification of possible regulation strategies when none are suggested while subsequent EoBF runs would allow subjects to focus on practicing identified strategies. But the study was not designed to substantiate such assumptions.

Given that regulation ability improved from session to session in the EoBF runs, additional TDs may further increase regulation capacity. It should be considered that our training was already more intensive than in most other reports on fMRI neurofeedback. Whether the cost of even more training sessions will be justifiable in the future will depend on the obtainable cost-benefit ratio of the particular application.

The regulation effect size in the transfer run of our study of *d* = 0.43 (95% conf. int. 0.23–0.64) was lower than reported by other groups for “explicit strategy” approaches. Zotev et al. (2011) reported, for example, an effect size of *d* ≊ 1 for the amygdala signal change in a transfer run without feedback. In that study, the transfer run was conducted directly after three training runs, all within a single session in 14 subjects. While it is unclear how significant the difference between these two effect sizes really is, clearly, our study does not provide evidence for a benefit of the “no instructed strategy” approach over the “explicit strategy” approach in terms of the effect size. We are currently collecting data to compare our findings directly with an “explicit strategy” approach using the same feedback setup and study design.

A controversial question within the context of neurofeedback with or without specific instructions is whether subjects are actually using regulation strategies as expected by the experimenters. In this context, it is particularly important whether subjects really regulate neural activity in the target region or manipulate the signal in other ways, for example through head motion or altered breathing. Most published studies do not address this issue in detail. However, despite the explicit instruction to regulate “using a purely mental strategy” and to “refrain from moving and to breath normally”, 13 of 32 subjects reported as their most successful strategies, next to mental approaches, also changes in body tension or breathing. We did not exclude these subjects from the analysis because permissible mental strategies such as relaxation or fear may well be reflected in physiological changes. An analysis of the recorded physiological data in 27 subjects, revealed that, while HR was elevated in the active regulation conditions, no difference between the “UP” and “DOWN” conditions was observed and no correlation with regulation capacity (Figure 6). With respect to respiration, however, we found evidence in a few subjects that this could have been a factor that contributed to the observed regulation capacity. While we do not see evidence for such physiology-related signals or motion in the group activation map (Figure 4), the individual subject map for subject 15, for example, (see Figure 5 bottom left) with widespread activation throughout the brain may be result of changes in respiration. This subject showed a relative change of RVT of 22%. At the same time, however, subject 14 does not show a global activation despite a change in relative RVT of 44%. On the group level, we found a trend for a difference in relative RVT between the “UP” and “DOWN” conditions and a correlation of this difference with regulation capacity (Figure 6). The sign of this difference is negative, which indicates “stronger” respiration in the “DOWN” condition. Stronger breathing without an increase in metabolic rate can lead to a reduction of blood flow to the brain (hyperventilation effect) and therefore a reduction in the BOLD signal, which is what we observe in the fMRI scan. These findings however, need to be interpreted with care for two reasons. First of all, RVT is at best a semi-quantitative measure when acquired with a respiration belt. While this is tolerable when used as a subject-level regressor, the numbers will be very noisy on the group level. Second, the correlation with regulation capacity is driven by a few outliers. Spearman rank correlation is far from significant (*R* = −0.183, *p* = 0.361). However, we conclude from the physiological data, that our measured effect size should be regarded as generous given that part of the effect may be due to changes in respiration.

On the other hand, the fact that the difference in subjective arousal ratings between “UP” and “DOWN” regulation strategies was positively correlated with regulation capacity (Figure 3C) is some evidence that regulation capacity is related to the expected functional role of the amygdala. We could also reproduce the previously reported negative correlation of the “Susceptibility to Anger” sub-score of the ECS with regulation ability in both post-training runs. And we found that subjects that are more agreeable/compassionate/understanding (NEO agreeableness) are less successful regulators. This may appear contradictory under the assumption that somebody who is more agreeable would be less susceptible to anger. However, these NEO and ECS subscores are not (negatively) correlated in our data. We could not confirm a negative correlation with the TAS-20 “Difficulty Identifying Feelings” sub-score.

As our attempts to identify patterns with respect to successful and unsuccessful regulation strategies were fruitless and given that this was not a major aim of this study, we are limiting our discussion on this topic here. Briefly, we believe that an analysis of the reported strategies was futile for two major reasons. First of all, our effect size and the number of subjects within our study were too small for this purpose, in particular given that no suggestions with respect to strategies were made. Second, the fact that a particular strategy worked for one subject does not mean that it will work for another. This means that statements such as “Strategy “A” worked well” need to be based on statistics in a larger group of subjects that all use the same strategy. Therefore, our recommendation to study the effectiveness of particular strategies is to pick two or three strategies and conduct an independent group study. An additional concern is that reported strategies may not reflect important learned, subconscious regulation mechanisms.

In conclusion, this study is the first to thoroughly investigate the ability of subjects to utilize fMRI neurofeedback without instructed or suggested strategies to learn bi-directional regulation of the amygdala. It is also one of the largest fMRI neurofeedback studies to date with 32 subjects included and five scanner days per subject. We found that subjects were able to utilize neurofeedback to learn to regulate the amygdala in the instructed directions after the training without feedback. The observed effect size of 0.43 was moderate and indicates that relatively large group sizes (*N* > 30) will be required to investigate differences between different neurofeedback approaches. Applying stricter exclusion criteria with respect to respiration changes would reduce this effect size further. Only marginal learning effects over sessions could be demonstrated. Whether “explicit strategy” approaches can outperform the “no instructed strategy” approach is currently under investigation.

## Author Contributions

All authors have contributed to the drafting of the manuscript, have agreed to its content, and have agreed to be accountable for all aspects of this work. MM, MJJ, SP, SB and MNS were involved in the conception and design of this study. MM, MJJ, DKM, EA, and RE were involved in data acquisition. All authors were involved in the analysis or interpretation of the data.

## Conflict of Interest Statement

The authors declare that the research was conducted in the absence of any commercial or financial relationships that could be construed as a potential conflict of interest. All authors acknowledge that this study benefitted from a collaboration with NeurInsight LLC (Albuquerque, NM, USA), a non-profit startup company owned by co-author S. Posse to share TurboFIRE with researchers free of charge.

## Footnotes

^1^While Dietrich et al. (2014) and Johnson et al. (2012) use the term “intermittent feedback”, we prefer the more descriptive term “end-of-block feedback”.

^2^http://www.fil.ion.ucl.ac.uk/spm/software/spm8

^3^http://www.translationalneuromodeling.org/team/lars-kasper/

^4^Additionally, note that that the study design does not allow for a pre-training transfer run because the transfer run requires the instruction to utilize the most successful strategies for left- and right-regulation identified during the training. This is, however, not relevant for the following argument.
