# Supplementary material for: Amygdala Regulation Following fMRI-Neurofeedback without Instructed Strategies
Source: Front Hum Neurosci. 2016 Apr 26;10:183. doi: 10.3389/fnhum.2016.00183 (PMC4844623; doi:10.3389/fnhum.2016.00183)

Supplementary Material

Regulating Amygdala Activity following fMRI-Neurofeedback without Instructed Strategies

Michael Marxen, Mark J. Jacob, Dirk K. Müller, Stefan Posse, Elena Ackley, Lydia Hellrung, Philipp Riedel, Stephan Bender, Robert Epple, and Michael N. Smolka^*^

*** Correspondence:** Corresponding Author: michael.smolka@tu-dresden.de

# Supplement S1: Exact wording of the regulation instructions to the subject (English translation below).

Anweisung zur Regulation

Ihre Aufgabe besteht darin, Ihre Gehirnaktivität durch rein mentale Strategien zu beeinflussen. Sie werden im Display ein Bild sehen wie unten. Die horizontale Position der Bälle repräsentiert die Aktivität in einer bestimmten Region des Gehirns. Der rote Ball repräsentiert die letzte Messung des Tomographen, während die lila Bälle die zurückliegende Aktivität darstellen. Je weiter unten der Ball desto weiter liegt die Messung zurück. Insgesamt wird die Aktivität der letzten 30s dargestellt.

Der Pfeil zeigt an in welche Richtung Sie regulieren sollen. Ein Balken bedeutet für Sie eine Pause. Sie brauchen nicht zu regulieren und können sich ausruhen.

Wie für alle Messungen im MRT möchten wir Sie allerdings bitten, insgesamt darauf zu achten, dass sie Bewegungen vermeiden und normal atmen. Außerdem ist es wichtig, dass die Augen geöffnet sind, um das Feedbacksignal immer sehen zu können.

Es wird drei Trainingssessions geben mit jeweils etwa 40 min. Zeit zur Regulation über drei Runs. Im letzten Run einer Session wird ein Feedback nur für 5s nach einer Regulationsperiode von 30s dargeboten.

Ihr Ziel sollte sein, nach dem Ausprobieren unterschiedlicher Strategien eine beste Regulationsstrategie zu finden/entwickeln, die Sie dann in einer weiteren Session nach dem Training auch ohne Feedback anwenden sollen.


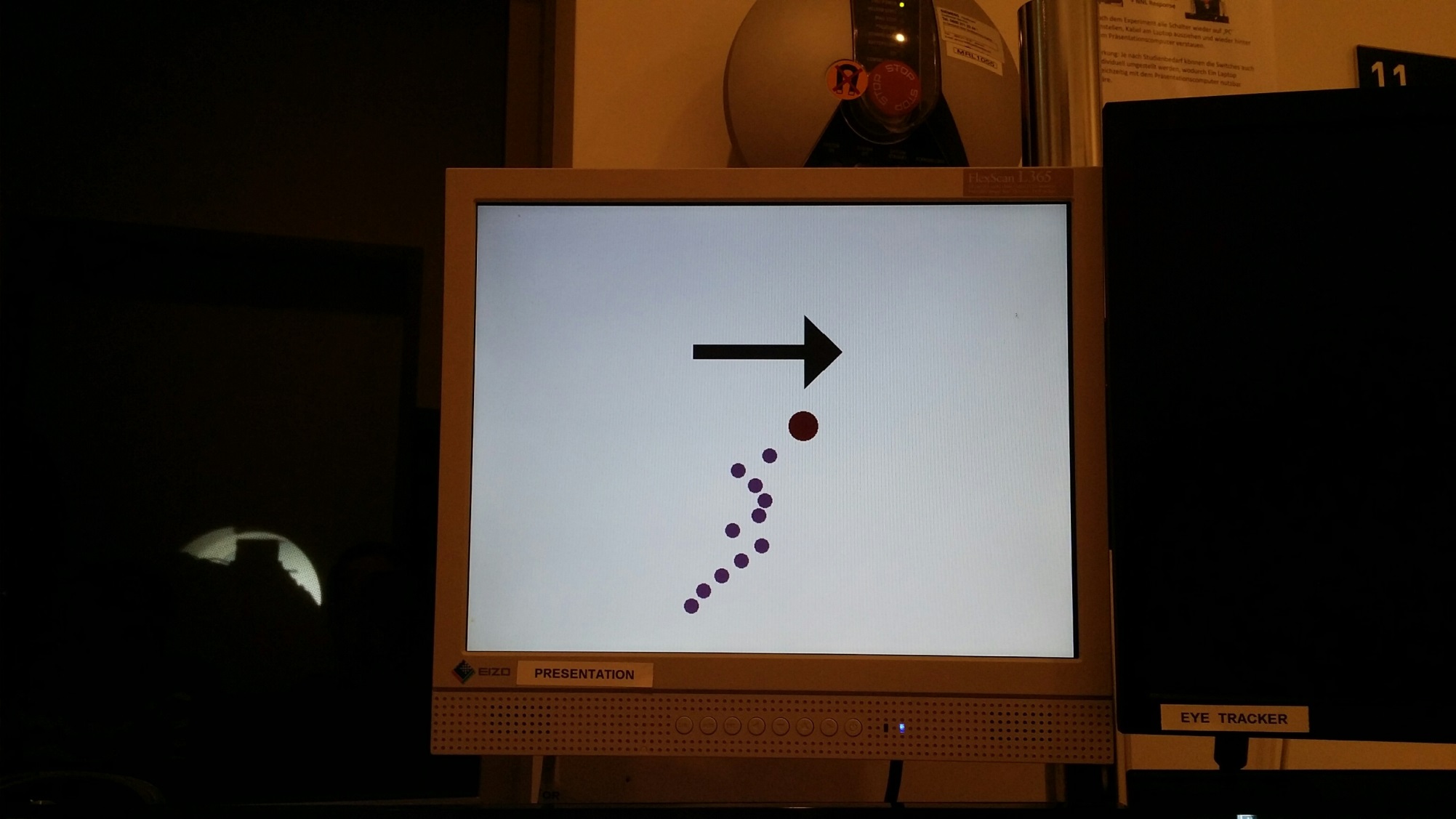


# Translation

Regulation Instructions

Your task is to influence your brain activity using a purely mental strategy. You’ll see a picture in the display as below. The horizontal position of the balls represents the activity in a certain region of the brain. The red ball represents the most recent measurement of the scanner, while the purple balls portray the previous activity. The further towards the bottom of the screen the ball appears, the older the measurement. In total, the activity of the last 30 seconds is displayed.

The arrow points in the direction you should regulate. A bar indicates you should rest. You don’t need to regulate and can have a rest.

As for all measurements in MRI, we would like to ask you to refrain from moving and to breathe normally. In addition, it is important for you to keep your eyes open so that you can always see the feedback signal.

There will be three training sessions, each of about 40 minutes with time for regulation spread over three runs. In the last run of a session, feedback is shown for 5 seconds after each thirty-second regulation period.

Your goal should be, having tried out various strategies, to find/develop an optimum regulation strategy, which you will then use also without feedback in a further session after the training.

Supplement S2: Questionnaire (German original and English translation below) with respect to the most successful strategies for each regulation direction. These strategies were employed during the post-training session.


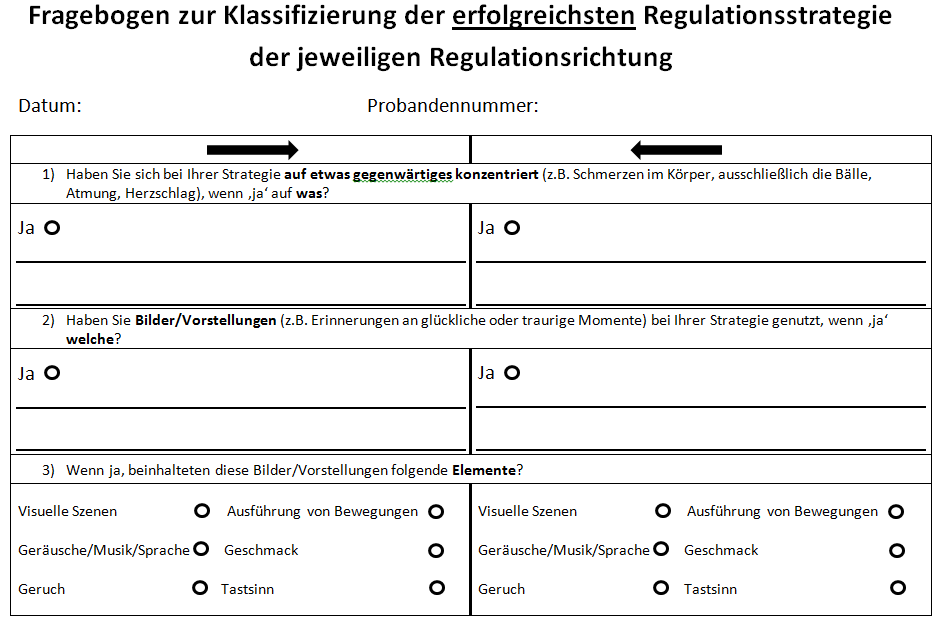


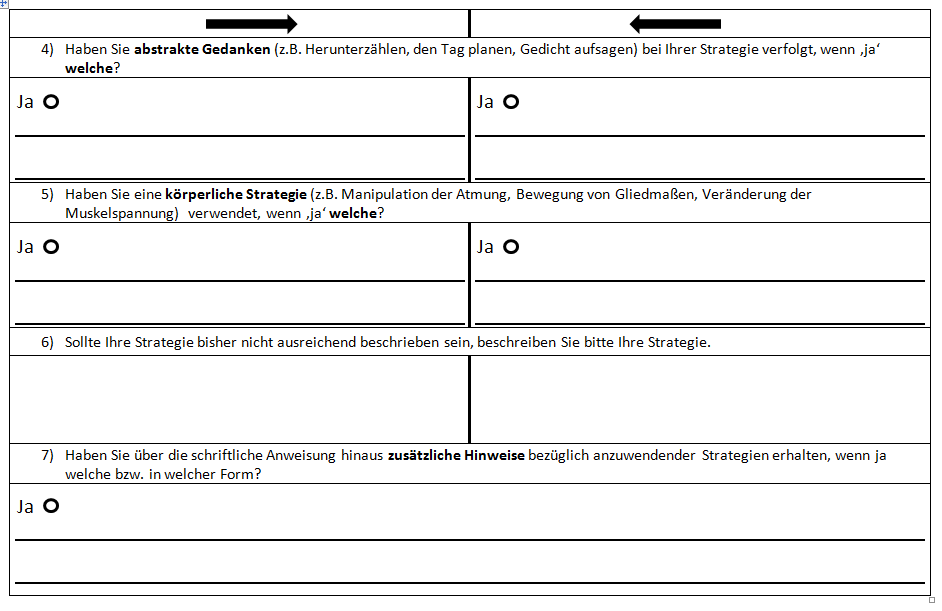


# Translation


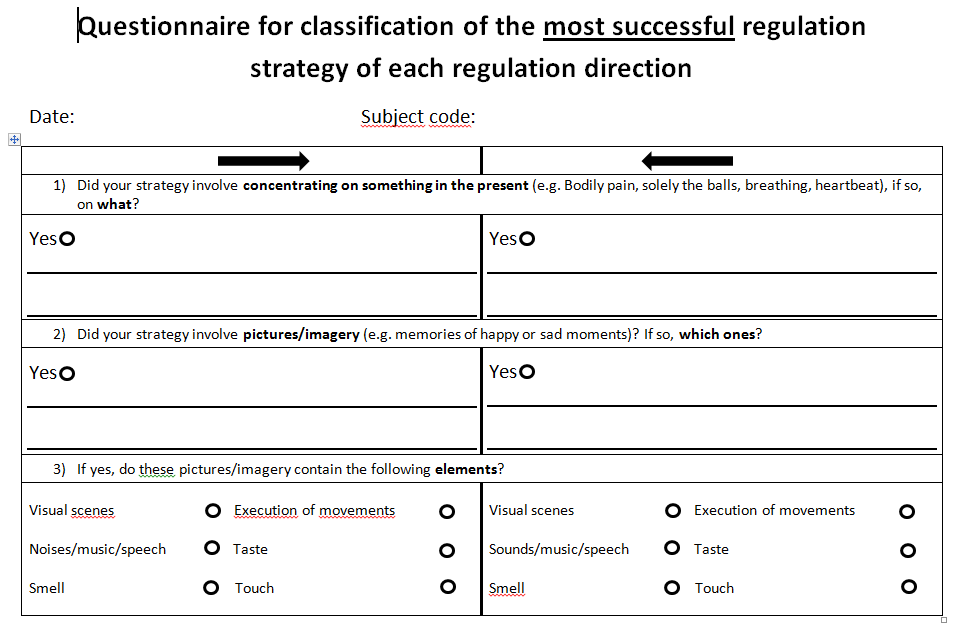

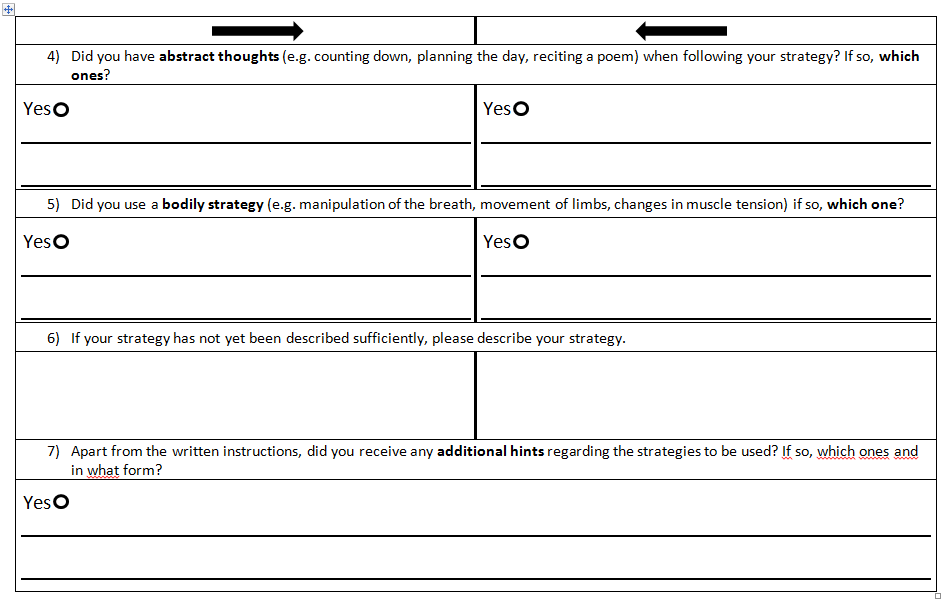

Supplement: Supplementary file 1 [file Table_1.docx]
